# Resonant Young’s Slit Interferometer for Sensitive Detection of Low-Molecular-Weight Biomarkers

**DOI:** 10.3390/bios15010050

**Published:** 2025-01-15

**Authors:** Stefanus Renaldi Wijaya, Augusto Martins, Katie Morris, Steven D. Quinn, Thomas F. Krauss

**Affiliations:** 1 School of Physics, Engineering and Technology, University of York, York YO10 5DD, UK; augusto.martins@york.ac.uk (A.M.); katie.morris2@york.ac.uk (K.M.); steven.quinn@york.ac.uk (S.D.Q.); thomas.krauss@york.ac.uk (T.F.K.); 2 York Biomedical Research Institute, University of York, York YO10 5DD, UK

**Keywords:** optical sensors, guided-mode resonance, Young’s interferometer, biosensors, limit of detection

## Abstract

The detection of low-molecular-weight biomarkers is essential for diagnosing and managing various diseases, including neurodegenerative conditions such as Alzheimer’s disease. A biomarker’s low molecular weight is a challenge for label-free optical modalities, as the phase change they detect is directly proportional to the mass bound on the sensor’s surface. To address this challenge, we used a resonant Young’s slit interferometer geometry and implemented several innovations, such as phase noise matching and optimisation of the fringe spacing, to maximise the signal-to-noise ratio. As a result, we achieved a limit of detection of 2.9 × 10^−6^ refractive index units (RIU). We validated our sensor’s low molecular weight capability by demonstrating the detection of Aβ-42, a 4.5 kDa peptide indicative of Alzheimer’s disease, and reached the clinically relevant pg/mL regime. This system builds on the guided mode resonance modality we previously showed to be compatible with handheld operation using low-cost components. We expect this development will have far-reaching applications beyond Aβ-42 and become a workhorse tool for the label-free detection of low-molecular-weight biomarkers across a range of disease types.

## 1. Introduction

Optical and nanophotonic biosensors offer applications in various domains and typically focus on protein biomarkers with applications in immunosensing [1,2]. Sensors utilising optical nanostructures that support guided mode resonances (GMRs) are particularly promising because they offer ease of light coupling and realisation with inexpensive components; simplicity, robustness, and affordability being particularly important for healthcare applications [3,4].

We note, however, that many high-performance sensor systems rely on costly light sources, spectrometers, or other read-out devices and often demand precise coupling setups [5]. In contrast, GMRs can be excited easily, even with LED light sources, and read out with a simple CMOS camera, yet are able to provide sensitive detection of protein biomarkers [6,7]. A GMR uses a periodic nanostructure that couples incident light into leaky waveguide modes, and the interplay between the Fabry–Perot resonance of the thin film and the Bragg resonance of the periodic structure then creates a sharp interference pattern at the resonance wavelength. This resonance shifts when target biomolecules bind to the sensor surface, altering the effective refractive index and enabling real-time, sensitive, and label-free detection [8].

For example, we have previously shown that a GMR-based sensor can be realised using low-cost nanoimprint lithography, while the readout instrument can be realised with very few optoelectronic elements and in a handheld configuration [9]; the modality can therefore meet the near-patient test requirement. We have also shown [10] that our approach affords quantification of protein biomarkers in the critical pg/mL range and that it offers multiplexing, which is important for reliable diagnosis based on multiple markers [11]. We suggest that our approach therefore offers a viable solution in the high performance/low-cost space.

Having previously demonstrated the detection of protein biomarkers of relatively high molecular weight (10s–100s of kDa), we now explore the detection of smaller molecules in this work. We focus on the 42 amino acid β-amyloid peptide (Aβ-42) (molecular weight 4.5 kDa [12]) as an exemplar because of its relevance for the early detection of Alzheimer’s disease [13], a progressive neurodegenerative disorder and leading cause of dementia, with substantial societal and economic costs [14]. The detection of plasma-based biomarkers, such as β-amyloid, is emerging as a promising approach for timely diagnosis and intervention [15]. Among these biomarkers, Aβ-42 is particularly relevant as it is linked to plaque formation in the brain which precedes cognitive decline [16]. However, its low molecular weight and low abundance in plasma (pg/mL) make detection extraordinarily challenging [17]. This motivates the development of new technologies that combine sensitivity and cost-effectiveness for practical deployment. Recent work on common-path interferometry [5,18] has demonstrated a promising approach to this problem by exploiting the interference between a low-quality (low-Q) transverse electric (TE) and a high-Q transverse magnetic (TM) mode, made to interfere via a Wollaston prism and an analyser. These systems have demonstrated promising detection limits but require complex and costly optical setups involving Wollaston prisms, polarizers, or high-precision alignment. While effective in a laboratory setting, such configurations are impractical for near-patient testing due to their high cost, fragility, and operational complexity. To bridge this gap, we present a novel GMR-based interferometric sensing modality that eliminates the need for additional optical components while achieving comparable or improved sensitivity.

We employ a resonantly enhanced interferometry approach based on Young’s slits [19], and implement a number of innovations to enable the very challenging detection of Aβ peptides; we carefully analysed the key features of the modality and significantly improved its noise performance. The resulting understanding helped us to significantly improve the detection limit and to reach the clinically relevant pg/mL range.

## 2. Results and Discussion

**Figure 1:**
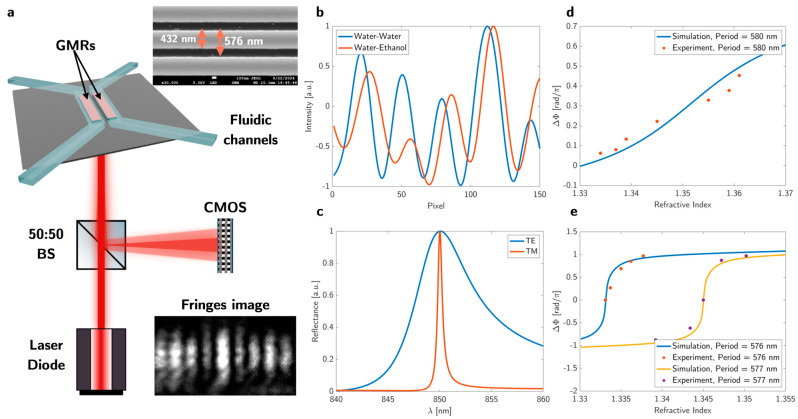
Schematic of the GMR sensor and sensing principle. (**a**) Each sensor contains two identical grating structures, one for the reference fluid and one for the analyte (further details on the sensor fabrication are explained in Appendix A). The two sensors are separated by a distance to form the fringe pattern. BS = beamsplitter; CMOS = complementary metal oxide semiconductor detector. The inset shows a scanning electron microscopy image of a fabricated GMR sensor. (**b**) When different fluids are placed on top of each sensor, the fringes shift (indicating a phase shift) according to the refractive index difference between each structure. (**c**) Simulation result of TE/TM mode. The TE/TM mode is excited when the E-field vector of the incident beam is oriented perpendicular/parallel to the grating vector. For the simulation, we used the following parameters: silicon nitride (n = 2) of 150 nm thickness as the grating layer on a glass (n = 1.45) substrate immersed in water (n = 1.3331). (**d**) Simulated and measured bulk sensitivity of the TE mode. (**e**) Simulated and measured bulk sensitivity of the TM mode. Both curves have different periods, so they can be measured at the same wavelength (further details on the measurement method are explained in Appendix B). ΔΦ shows the phase difference between the signal and reference channel. For the measurement shown with the blue line, we used DI water (n = 1.3331) as our reference. On the yellow line, we used a mixture of DI water and ethanol (n = 1.3459) as our reference to show that we can tune the dynamic range by changing the period to match the analytes’ refractive indices.

In the classical Young’s interferometer, monochromatic light passing through two slits generates an interference pattern of bright and dark fringes, indicative of the phase difference between the corresponding light waves. Traditionally, the sensitivity of a Young’s slit interferometer is controlled by the thickness of the analyte channel, creating phase differences that result in constructive and destructive interference [20,21]. However, by replacing the slits with GMR reflectors, we can exploit the steep phase gradient occurring near a resonance, thereby increasing the sensitivity by at least an order of magnitude. The key difference lies in the fact that the resonance of one GMR shifts due to the presence of the analyte, while the other remains unchanged. This creates a phase difference between the two resonant beams, which then interfere with each other. The resulting interference pattern encodes the analyte-induced changes, allowing us to extract sensitive detection information. Furthermore, this arrangement operates in reflection mode, which allows us to position the fluidics on top of the sensor while illuminating from below, as shown in Figure 1a. Reflection mode eliminates the need for light to pass through the fluidic section, which can easily cause phase disturbances. By changing the refractive index on top of one GMR reflector, it exhibits a phase difference between the sensors, which leads to a shift in the interferogram, as shown in Figure 1b.

A GMR grating exhibits birefringence by supporting orthogonally polarised modes. When the magnetic field of the incident light is polarised perpendicular to the grating vector, along the grating grooves, it excites the TM mode, while the electric field polarised perpendicular to the grating vector excites the TE mode. The symmetry of the TM mode suppresses out-of-plane radiation, akin to a bound state in the continuum (BIC), which results in a higher quality factor compared to the TE mode (Figure 1c) and correspondingly allows us to achieve a lower limit of detection. 

The GMR sensor was designed for a wavelength of 850 nm to match the wavelength of a diode laser. To evaluate its performance, we conducted a series of tests using ethanol solutions of varying refractive indices. From the corresponding response (Figure 1d,e), we determined a phase sensitivity of S = 48 rad/RIU for the TE mode and S = 1560 rad/RIU for the TM mode in the linear steep section at the centre of the resonance, which confirms our choice of TM mode (further details are explained in Supplementary Information S1 and S2).

### 2.1. Approaches to Noise Reduction

**Figure 2:**
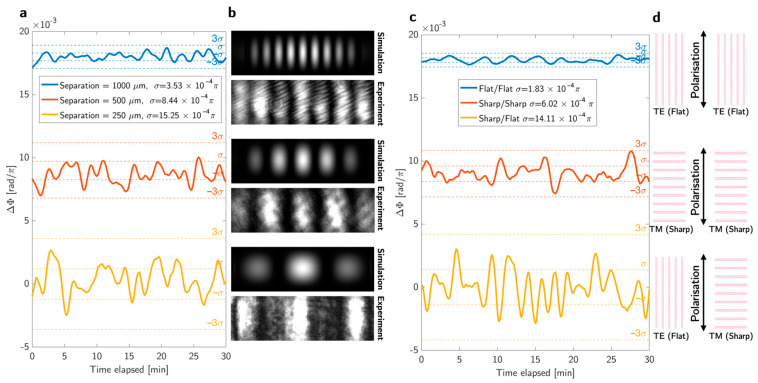
Different approaches to minimise noise. (**a**) Noise reduction by slit spacing variation. By separating the slits further, more fringes appear in the field of view that afford a better determination of the fringe pattern position, thus reducing noise. (**b**) Corresponding simulation and experimental fringe patterns as a function of slit separation. (**c**) Noise reduction using intrinsic phase-noise matching, for interference between the “sharp” phase response of the TM mode against the “flat” phase response of the TE mode. The top curve describes flat/flat, the middle curve sharp/sharp and the bottom sharp/flat. As expected, the sharp/flat exhibits the highest noise due to the phase mismatch. Of the two phase-matched scenarios, the flat/flat approach exhibits slightly lower noise, but since it also has the lowest phase sensitivity, the overall signal/noise ratio is best for the sharp/sharp approach. (**d**) Schematics of different approaches corresponding to the curve. Vertical gratings indicate TE (flat) mode and horizontal gratings indicate TM (sharp) mode.

A highly sensitive sensor is inherently susceptible to noise. Therefore, achieving a low limit of detection (LOD) necessitates effective noise reduction. We explored two strategies to this end, namely the separation of the Young’s slits and the principle of intrinsic phase-noise matching [22]. The first strategy is to optimise the spacing of the slits. The slit spacing determines the spatial distance between the fringes. The distance between the fringes is inversely proportional to the slit spacing; therefore, increasing the spacing results in a shorter distance between fringes, resulting in a higher number of fringes within the field of view (FOV). In turn, a larger number of fringes reduces the ability to determine the position of each fringe, because it is defined by fewer pixels on the camera. We explored this trade-off and found that a larger number of fringes improves the noise, as clearly shown in Figure 2a. The rationale is that, by increasing the number of fringes being recorded (as shown in Figure 2b), the determination of the fringe period becomes more accurate and allows us to better determine the shift; it is therefore the position of the ensemble of fringes rather than the position of individual fringes that determines the resolution.

The second strategy of intrinsic phase-noise matching relies on the understanding that both the signal and the noise response are amplified in the steep section of the phase curve (Figure 1d,e). Typically, the interference happens between two signals, where one is resonantly enhanced and the other is chosen to have a constant phase response [22,23]. In the context of our geometry, this would be the case for one of the reflective Young’s slits being on resonance, with the other one being a gold mirror. However, this means that the two interfering signals exhibit different phase responses to the environmental noise, such as wavelength and temperature fluctuations. In that case, upon interference, the overall system noise increases, because the resonant channel responds more strongly to the noise than the non-resonant channel. To address this discrepancy, we use the same resonance in both channels, such that the noise intrinsically cancels out; if there is the same noise in both channels, there is no interference, because interference only records a phase difference, but not an absolute phase.

Figure 2c illustrates the observed noise over 30 min between the various interference combinations; we refer to the resonant phase response as “sharp” because of its sharp phase response and to the non-resonant phase response as “flat”. All configurations have the same slit separation of 875 μm, with the orientations as shown in Figure 2d. Starting from the bottom, the non-matched approach (sharp/flat) exhibits higher noise than both others. While the flat/flat approach exhibits slightly lower noise than the sharp/sharp approach, it also does not benefit from the resonant enhancement of the desired phase response. Recall that, in Figure 1, we show that the “sharp” phase response is >30 times steeper than the “flat” response, enhancing the sensitivity by the same factor. The superiority of the sharp/sharp interference is also evident from the limit of detection. We measured the noise by keeping identical fluids flowing on top of both sensors and recording the phase shift over a 30 min period. The standard deviation was calculated, showing that the phase-matched configurations (sharp/sharp and flat/flat) exhibited lower noise than the sharp/flat configuration. The most important parameter in characterising a sensor is the LOD = 3 σ/S [8], which is the minimum resolvable change that can be detected by the system. We achieved values of 8.5 × 10^−6^ RIU (sharp/flat), 3.6 × 10^−6^ RIU (sharp/sharp), and 35.9 × 10^−6^ RIU (flat/flat) for the three configurations. The 3σ value, which is three times the standard deviation of the blank signal, is used because it balances low false-positive rates with sufficient sensitivity to detect genuine signals [24]. Since the sharp/sharp has a higher sensitivity to desired phase changes by a factor of >30 compared to the flat/flat approach, it clearly shows the best performance. By combining both noise reduction approaches using the 1000 μm separation and sharp/sharp configuration, we achieved an LOD = 2.9 × 10^−6^ RIU.

### 2.2. Protein Sensing

Finally, we applied the optimised sensor to the problem of detecting biomarkers for Alzheimer’s disease, focusing on Aβ-42, a peptide that aggregates in the brain and that can cross the blood–brain barrier, most likely because of its small size. To illustrate the problem, when we first started this work, we used our standard GMR assay; this assay can detect standard protein biomarkers such as troponin or C-reactive protein down to levels of 10 pg/mL [10]. When using the same assay for Aβ-42, the best we were able to achieve was 20 ng/mL. This comparison clearly highlights the need for improvement. 

**Figure 3:**
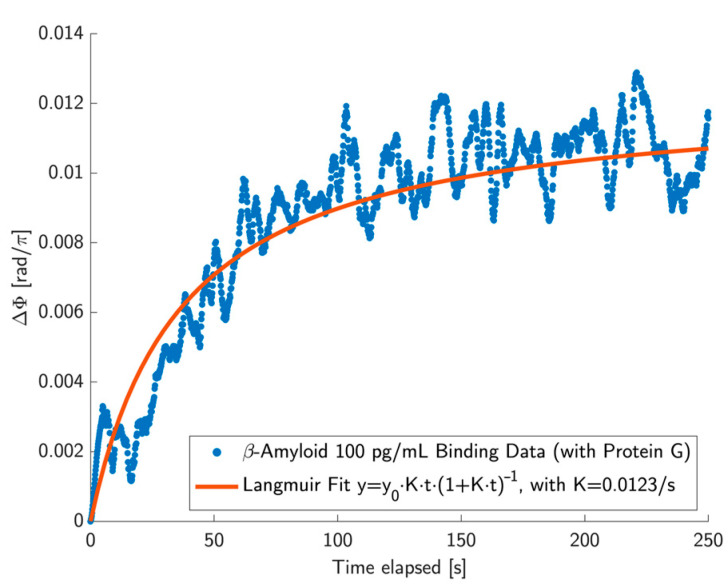
Binding of monomeric Aβ-42 on optimised Young’s slit interferometer. Langmuir isotherm fitting of β-Amyloid at a concentration of 100 pg/mL demonstrates that the β-Amyloid binding follows the expected trend of antibody-ligand association (further details on the surface chemistry are explained in Appendix C).

Using the optimised Young’s slit approach, we were able to achieve much better performance, as demonstrated by the detection of 100 pg/mL Aβ-42 in PBS buffer (pH 7.4) over the course of 150 s (Figure 3). By following the Langmuir binding curve, we ensured that we observed actual binding rather than simple physisorption [5], with the K-value indicating the rate of detection. For details of the protocol, please refer to the Appendix section. By achieving a limit of detection (LOD) in the pg/mL range, our sensor enters the clinically relevant range. The levels of Aβ-42 that need to be detected are the subject of intense research, so it is difficult to provide clear-cut numbers. Nevertheless, a range of 36 pg/mL to 140 pg/mL has been reported for Alzheimer’s patients in various studies [17], which is clearly in the range of our sensor. Moreover, multiple markers need to be detected to properly pinpoint the disease, which we have already shown for our sensor modality [10]. We have also shown that our modality can be realised as a handheld instrument [9], thereby meeting a key criterion for its widespread deployment. In this context, it is important to note the simplicity of the Young’s slit approach and the mitigation of environmental noise enabled by the intrinsic phase-noise matching we implement here.

## 3. Conclusions

In conclusion, we have simplified and improved a resonant Young’s slit interferometer arrangement and have demonstrated its ability to detect low-molecular-weight molecules. Specifically, we have shown the detection of the Aβ-42 peptide, a molecule of 4.5 kDa molecular weight indicative of Alzheimer’s disease, in vitro. The key improvements have been to (a) employ the idea of phase noise matching, which intrinsically subtracts the noise between the two interfering channels, and to (b) optimise the fringe spacing, where we found that fidelity in terms of determining the phase shift increases with the number of fringes recorded. Together, we achieved a limit of detection of 2.9 × 10^−6^ RIU and <100 pg/mL, which is in the clinically relevant concentration of 36–140 pg/mL [17]. Detecting Aβ-42 in vitro with this approach now sets the stage for downstream studies in more complex biofluids, such as plasma, which will require a suitable antifouling strategy, e.g., the use of PEG layers [10]. Additionally, the multiplexed detection of several markers, such as Aβ-40 and tau [25] in addition to the Aβ-42 shown here, will be essential to increase the fidelity of the diagnosis.

We suggest that the simplicity and cost-effectiveness of our system make it a promising candidate for the wider application of biosensing. We recognise that other modalities have already been demonstrated for the detection of Alzheimer biomarkers [26,27,28,29], some of which have shown impressive performance. Nevertheless, as we have recently pointed out [27], the suitability of a biosensing modality for healthcare applications depends on many factors, including scalable manufacturing, reproducibility, user-friendliness and cost, to name but a few. In this context, it is too early to decide which modality is most advantageous for solving this important problem, which justifies the search for alternatives [27,28,29]. Our GMR-based sensor modality can be readily implemented in a handheld format [9], the grating chips can be made by nanoimprint lithography [9], and interferometry is possible with a filtered LED [22]. Together with the ability to detect small peptides that we have now demonstrated, this work shows clear potential to contribute towards the label-free biosensing of a broad class of low-molecular-weight markers. In the context of Alzheimer’s disease, downstream developments could contribute towards early-stage diagnosis, and personalised treatment interventions years before clinical symptoms become apparent.

## Data Availability

The data that support the findings of this study are available from the corresponding author upon reasonable request.

## Supplementary Materials

The following supporting information can be downloaded at: https://www.mdpi.com/article/10.3390/bios15010050/s1, S1. Bulk Sensitivity Measurement; S2. Limit of Detection Measurements Using TM Mode; S3. Microfluidic System Integration; S4. Optical Setup; S5. Data Processing.

## Appendix A. Sensor Fabrication

The chip fabrication process involved the following general procedures: A commercial wafer, consisting of a 150 nm thick film of silicon nitride (Si_3_N_4_) on a 500-μm glass substrate (Silson, Southam, UK), was diced into 15 × 15 mm^2^ pieces. These pieces were cleaned by sonication in acetone (ACE) for 10 min, rinsed in isopropanol (IPA), and subsequently cleaned in O_2_ plasma. For Electron Beam Lithography (EBL), we spun 380 nm of ARP-13 resist on the substrate and used ARPC as charge dissipation layer. The pattern was inscribed using a Raith GmBH 50 kV Voyager EBL system. After the exposure, the charge dissipation layer was rinsed in DI water and the resist was developed in xylene at room temperature. The pattern was transferred into the silicon nitride layer via plasma-based Reactive Ion Etching (RIE) using a gas mixture of oxygen (O_2_) with a 2 sccm flow rate and trifluoromethane (CHF_3_) with a 58 sccm flow rate. Initially, the RIE chamber was cleaned with hydrogen plasma and the pressure reduced to below 10^−5^ mbar. The etching process was conducted at a voltage of 360 V, translating to approximately 41 W power in our system for 7 min. After etching, the remaining resist was removed by gentle sonication in 1165 Microposit Remover for 10 min, followed by cleaning with ACE, IPA, and plasma ashing.

To form the channels, a mould was first created using 3D printing at the highest available resolution of 50 μm. Once the mould was ready, polydimethylsiloxane (PDMS) was prepared by mixing the elastomer and curing agent in a 7:1 ratio. The mixture was then placed in a desiccator for at least 30 min to remove air bubbles. After degassing, the mixture was poured over the mould and placed back in the desiccator to eliminate any remaining air bubbles. The sample was then baked at 60 °C for a minimum of 8 h to ensure complete cross-linking of the polymers. Following curing, the PDMS was carefully removed from the mould, and holes for the tubing are punched. The tubes were then installed, and the channels attached to the sensor. A clamp was used to secure the sensor and channels together (further details are explained in Supplementary Information S3).

## Appendix B. Optical Setup and Data Processing

The setup for this measurement primarily includes a light source, beam splitter, polarizer, and camera. An 850 nm diode laser (Thorlabs CPS850V, Newton, MA, USA) serves as the light source. A pellicle beam splitter was used to eliminate ghost images. The light was reflected by the sensor and then passed through a linear polariser, which is essential for selecting the correct mode and removing scatterings. The interferogram is captured by a CMOS camera (Edmund Optics EO−5012M, Barrington, IL, USA), and fast Fourier transform−based post−processing was performed with a custom MATLAB script (further details are explained in Supplementary Information S4 and S5).

## Appendix C. Surface Chemistry

The sensor surface is initially cleaned using acetone (ACE) and isopropanol (IPA), followed by treatment in a plasma asher at 100% power for 30 min. This step removes contamination from previous measurements without etching the sensor, unlike cleaning with piranha solution. Next, the sensor was coated with dopamine (Sigma−Aldrich, St. Louis, MO, USA) with a concentration of 2 mg/mL to promote protein adhesion. Following this, the microfluidic channels were clamped in place. The process begins with flowing Neutravidin with a concentration of 250 µg/mL (Thermo Fisher Scientific, Waltham, MA, USA), a deglycosylated form of avidin known for its high affinity for biotin, to ensure the binding of biotinylated molecules. This is followed by the introduction of biotinylated Protein G with a concentration of 5 µg/mL (Pierce Biotechnology, Waltham, MA, USA). Protein G is an antibody binding protein expressed in certain strains of bacteria. It ensures the correct orientation of the antibodies, so the antigen binding domain is accessible. The chosen antibody (GenScript Biotech, clone 25G13, catalogue number V28701, Piscataway, USA) was then introduced using a syringe pump set to a flow rate of 75 μL/min. The antibody was mouse monoclonal but produced in vitro without animal derived products. A 10 min Phosphate-buffered saline (PBS) washing step was performed to remove any unbound proteins. This was followed by a 15 min application of a casein-blocking buffer (Sigma-Aldrich), diluted 10×, to block any remaining nonspecific binding sites. Additional PBS washing continues until a stable phase response is achieved, which can take up to 30 min. Once stability was confirmed, Aβ-42 (Eurogentec, Seraing, Belgium) was flowed through the system in PBS buffer (pH 7.4) to measure refractive index changes.
