# Supplementary material for: Resonant Young’s Slit Interferometer for Sensitive Detection of Low-Molecular-Weight Biomarkers
Source: Biosensors (Basel). 2025 Jan 15;15(1):50. doi: 10.3390/bios15010050 (PMC11763694; doi:10.3390/bios15010050)
Supplement: Supplementary file 1 [file biosensors-15-00050-s001.zip › biosensors-3317420-supplementary.pdf]

SUPPLEMENTARY INFORMATION

Resonant Young's Slit Interferometer for Sensitive Detection of Low-Molecular-Weight Biomarkers

Stefanus Renaldi Wijaya[1], Augusto Martins[1], Katie Morris[1], Steven D. Quinn[1,2], and Thomas F. Krauss[1]

[1]School of Physics, Engineering and Technology, University of York, York, YO10 5DD, U.K.

[2]York Biomedical Research Institute, University of York, York, YO10 5DD, UK.

Keywords: *Optical sensors, guided-mode resonance, Young's interferometer, biosensors, limit of detection*

S1. Bulk Sensitivity Measurement

To test the sensor's performance in the real world compared to the simulation, we need to test it using fluidics with an increasing refractive index within the resonance range. This is achieved by flowing ethanol solutions of known refractive index over the sensor and measuring the corresponding response. A series of arbitrary ethanol solutions are prepared, and their refractive index is measured using a refractometer. From these responses, the phase curve is obtained, and the sensitivity can be calculated by fitting the steepest part of the phase curve.

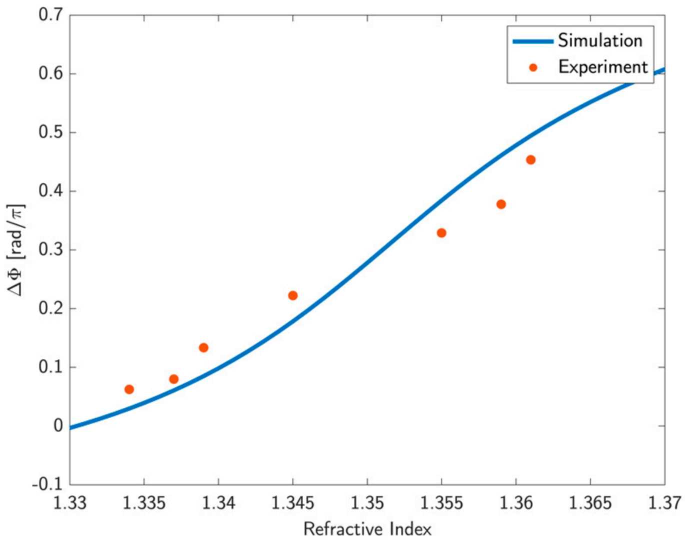

Figure S1. Bulk sensitivity measurements using the TE mode.

Figure S1 shows a strong agreement between the simulated and measured phase curve shapes, resulting a sensitivity of 48 rad/RIU. To align the position of the phase curve, we adjusted the period to fine-tune the resonance wavelength in the experiment. This adjustment ensures an overlap between the experimental data and the simulation. This has to be done due to various factors, such as the discrepancy between the advertised and actual thickness of the high-index layer, fabrication uncertainty, and angle of incidence. By keeping the same filling factor and only changing the period, only the whole phase curve will be shifted without changing the shape of the phase curve.

S2. Limit of Detection Measurements Using TM Mode

Although the previous section reveals good agreement between the simulated and measured data, the resulting phase sensitivity is too low to measure miniscule changes in refractive index, for example, during protein binding. In order to do this, we need a higher-Q resonance, which is supported by the TM mode, as shown in Figure S2, resulting a sensitivity of 1560 rad/RIU.

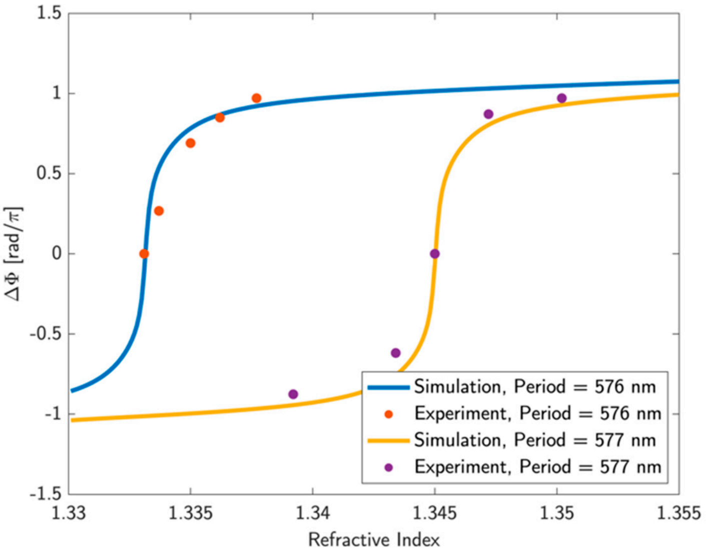

Figure S2. Bulk sensitivity measurements using the TM mode with two different periods, highlighting the trade-off between the dynamic range and the sensitivity.

To measure the noise level, we flow same analytes in both signal and reference channel for 30 minutes and measure the phase difference between them. We achieved the lowest noise level of 1.5 mrad, and by combining the sensitivity and the noise level we can measure the LOD = 3σ/S = 2.9 x 10^{-6} RIU.

S3. Microfluidic System Integration

To form the channels, a mould is first created using 3D printing at the highest available resolution of 50 µm. We found that the resolution of our 3D printer (Formlabs 3) is high enough to form two 300 µm-wide channels with 150 µm separation. This method is much faster and cheaper than the common way to form microfluidics channels using thin film such as SUEX (DJ Microlaminates, US).

Once the mould is ready, PDMS is prepared by mixing the elastomer and curing agent in a 7:1 ratio. The mixture is then placed in a desiccator for at least 30 minutes to remove air bubbles. After degassing, the mixture is poured over the mould and placed back in the desiccator to eliminate any remaining air bubbles. The sample is then baked at 60°C for a minimum of 8 hours to ensure complete cross-linking of the polymers. Following curing, the PDMS is carefully removed from the mould, and holes for the tubing are punched. The tubes are then installed, and the channels are attached to the sensor. A clamp is used to secure the sensor and channels together, and it can also be mounted to the sample holder.

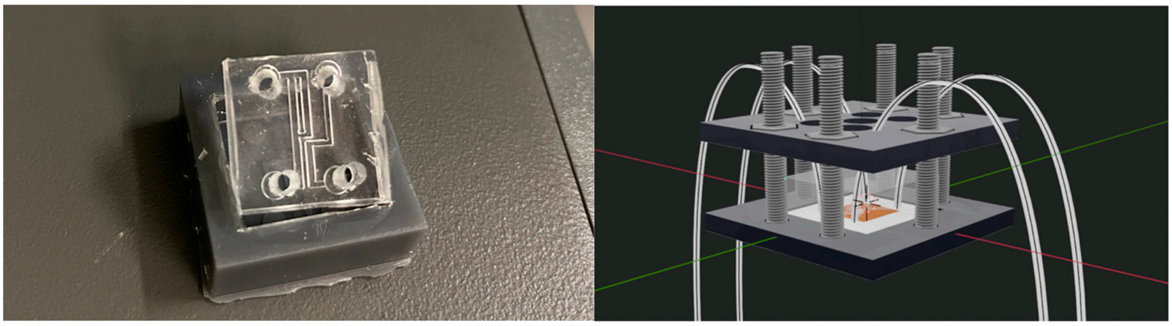

Figure S3. Fabrication result of the microfluidic channels (left) and 3D-render of the clamping system (right)

S4. Optical Setup

The setup for this measurement primarily includes a light source, beam splitter, polarizer, and camera. An 850 nm diode laser serves as the light source. A pellicle beam splitter is used to eliminate ghost images. The light is reflected by the sensor and then passed through a linear polariser, which is essential for selecting the correct mode and removing scatterings. The interferogram is captured by a CMOS camera, and the system is controlled using a custom Python script. The resulting data is saved in a .csv file for subsequent phase analysis.

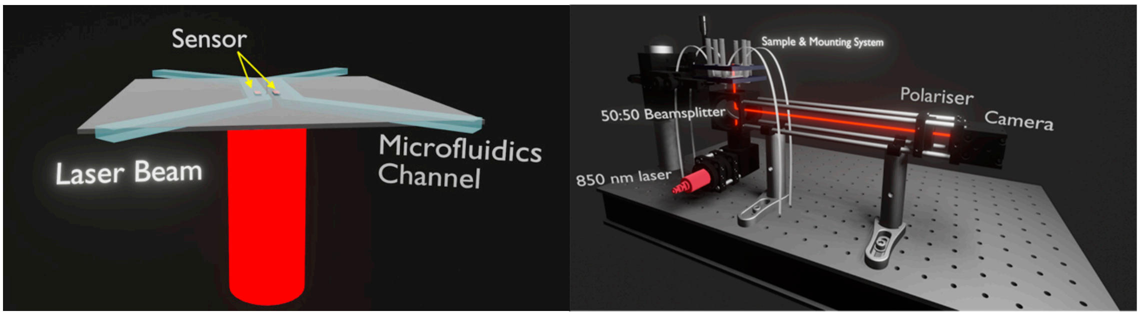

Figure S4. Arrangement of input light, sensors, and microfluidic channels (left) and optical setup for the measurements (right). The clamping system is mounted with a Thorlabs sample holder, allowing adjustment in x- and y- direction. The z-direction can be controlled using the stage, and the pitch and yaw can be controlled using a kinematic mount. The laser beam goes through the beamsplitter and is reflected by the sensor, before bounced by the beamsplitter towards the camera through a linear polariser.

S5. Data Processing

The video output from the camera is processed by extracting each frame using a custom Python script. A Region of Interest (ROI) is manually selected and applied to all frames. Each ROI is then Fourier transformed, and a threshold is set to omit all pixels with brightness below this value. The filtered image is obtained by performing an inverse Fourier transform, resulting in a clean image. Each ROI from every frame is sliced by pixel row, and a sine function fit is applied to extract the phase or position of the fringe pattern.

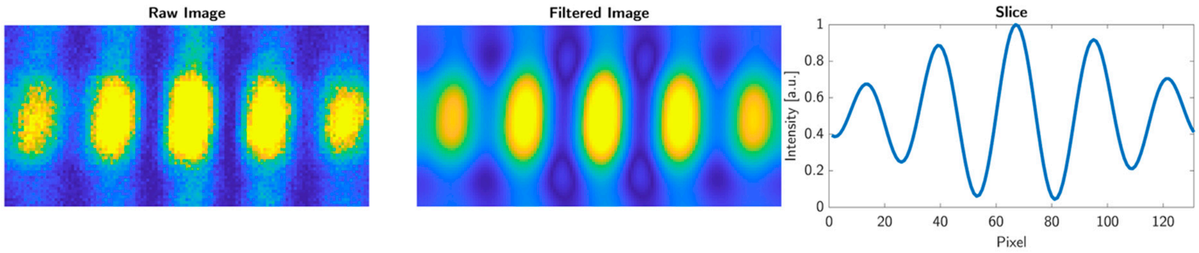

Figure S5. Fourier filtering of interferogram. Left image shows the raw image captured by the camera, centre image shows fourier-filtered of the image, right shows the average value of the interferogram.

By plotting the phase development over time, it is possible to observe when different fluids with varying refractive indices enter the sensor. To reduce computational noise over time, a Savitzky-Golay filter is applied to the response signal. This digital signal processing technique smooths and reduces noise by employing a moving polynomial fit to approximate the signal. It effectively balances noise reduction while preserving important features. The order and window width of the filter can be adjusted to optimise performance. This filtering approach is combined with Fourier filtering to mitigate high-frequency fluctuations, functioning similarly to a combination of a low-pass filter and a median filter.
